# Implementation of an electronic hospital outbreak detection system

**DOI:** 10.1186/2047-2994-4-S1-O54

**Published:** 2015-06-16

**Authors:** M Behnke, LA Peña Diaz, B Piening, G Pilarski, P Gastmeier, C Schröder

**Affiliations:** 1 Institute of Hygiene and Environmental Medicine, Charité – University Medicine Berlin, Berlin, Germany

## Introduction

The early detection of infectious disease outbreaks plays a key role for infection control professionals (ICPs) in a hospital in order to initiate infection control measures. Hospital information systems do not offer ordinarily support in this field. We identified the need for an easy to use, web-based, electronic system to detect unusual clusters of positive microbiology results.

## Objectives

To implement a hospital-based user-friendly microbial pathogen cluster detection system.

## Methods

The Institute for Hygiene in a 3,000 beds tertiary hospital implemented a software to detect clusters of pathogens (CP). The microbiology lab sends all reports to our server which inserts them into a data warehouse (DW). We implemented 2 cluster detection algorithms (CDA) into CP. They alert high numbers of lab results in a time series. The first algorithm is a cumulative sum (CUSUM) strategy. The second one calculates a confidence interval (CI). The user defines rules specifying the species of the microorganisms, the wards to look at and the time intervall for the detection resolution. CP scans the DW. Depending on the defined rules, the CDA are executed. Detection results are presented to the user in charts and tables. To validate the system, we compared 3 conventional documented outbreaks against the findings of the algorithms.

## Results

First, the conventional documentation shows a vancomycin resistant Enterococcus (VRE) outbreak on an ICU, first isolate found at 4/4/2014, totaling 3 cases. CI detected the first positive lab result at the same day and counts the 3 cases. CUSUM found the first result 6 days earlier and counts in total 7 isolates. Secondly, a multidrug resistant Acinetobacter baumanii outbreak including 3 cases, started at 10/14/2014. CI recognized the first positive isolate at 10/02/2013 and counted 4 cases. CUSUM had the same results as CI. Third, 10 cases of Carbapenem-resistant Klebsiella pneumoniae occurred on an ICU, first isolate at 02/10/2013. CI found 11 cases, starting at 02/04/2013.

## Conclusion

The algorithms detected all documented clusters. They alerted in many cases earlier than the ICPs. Further adjusting of alerts is necessary to avoid mis-alarming. In near future the ICPs in our hospital will get a useful intranet tool for the early detection of clusters of pathogenes.

## Disclosure of interest

None declared.

